# Effects of selected socio-demographic characteristics on nutrition knowledge and eating behavior of elementary students in two provinces in China

**DOI:** 10.1186/s12889-017-4580-5

**Published:** 2017-07-14

**Authors:** Ling Qian, Fan Zhang, Ian M. Newman, Duane F. Shell, Weijing Du

**Affiliations:** 1 Department of Guidance and Training, China Center for Health Education, Building 12, Block 1 Anhua Xili, Andingmenwei, Beijing, 100011 China; 2 Department of Epidemiology, School of Public Health and Management, Chongqing Medical University, Yixueyuan Road No. 1, Yuzhong, Chongqing, 400016 China; 3 Department of Educational Psychology, University of Nebraska-Lincoln, PO Box 880345, Lincoln, NE 68588-0345 USA; 4 Department of Guidance and Training, Chinese Center for Health Education, Building 12, Block 1 Anhua Xili, Andingmenwei, Beijing, 100011 China

**Keywords:** Cluster analysis, Socioeconomic factors, Demographics, Parent education, Elementary school children, BMI, Nutrition knowledge, Nutrition behavior, School-based, China

## Abstract

**Background:**

National and international child health surveys have indicated an increase in childhood obesity in China. The increase has been attributed to a rising standard of living, increasing availability of unhealthy foods, and a lack of knowledge about healthy diet. The objective of this study was to assess the effect of selected socio-demographic characteristics on the BMI, nutrition knowledge, and eating behavior of elementary school children.

**Methods:**

Multistage stratified cluster sampling was used. Information on demographics, nutrition knowledge, and eating behavior was gathered by means of questionnaires. The schools’ doctors provided the height and weight data. The study was set in one economically advantaged and one economically disadvantaged province in China. The participants were Grade 3 students, ages 8–10 years (*N* = 3922).

**Results:**

A cluster analysis identified four socio-demographic variables distinguished by parental education and family living arrangement. A one-way ANOVA compared differences among the clusters in BMI, child nutrition knowledge, and child eating behavior. Students in the cluster with lowest parent education level had the lowest nutrition knowledge scores and eating behavior scores. There was no significant benefit from college education versus high school education of parents in the other three clusters. BMI was not affected by parent education level.

**Conclusion:**

The nutrition status of elementary school age children will benefit most by increasing the general level of education for those adults who are presently least educated.

## Background

Childhood obesity, a risk factor for many diseases, is one of the world’s most serious public health challenges [1, 2]. Low- and middle-income countries report the fastest increase in overweight young children [1]. In 2010, Song et al. estimated the prevalence of obesity for urban Chinese children was 8.1% [3]. A meta-analysis of 20 studies published between 2009 – 2014 estimated China’s child obesity rate to be 10.4% [4]. In urban areas in China, the diet of children has changed as a result of globalization and the introduction of less healthy foods [5]. At the same time it has become clear that nutrition knowledge is low and many eating behaviors are unhealthy [6, 7].

A search of the two largest Chinese research databases—China National Knowledge Infrastructure (CNKI) [8] and Wei’pu (VIP) [9] —yielded 27 papers describing primary school children’s eating behavior and nutrition knowledge published in the last 15 years. The papers were published in 15 different Chinese journals and described samples from 14 different provinces. Two studies used the World Health Organization’s (WHO) Global School Health Status Survey instrument [10, 11], three studies used an instrument designed by the China Centers for Disease Control and Prevention (CDC) [10, 12, 13], and the remaining papers used investigator-designed survey instruments. Two papers compared the knowledge, attitudes, and practices (KAP) of normal-weight students and overweight/obese students [14, 15]. The papers all varied in scope and focus, and the results reported were usually descriptive.

This review suggested that family socio-demographic characteristics contributed to obesity, nutrition knowledge, and eating behaviors of primary school children. For example, Li, et al. [16] found that children from higher economic status provinces had higher obesity rates. Gu, et al. [17] found that parents with more education were more aware of their children’s health and weight. Other studies found that children in three-generation families with grandparents cooking were more likely to be overweight [18, 19]. One study [11] noted that body image affected eating behavior: Children who saw themselves as normal weight had better eating behaviors than children who saw themselves as over- or under-weight. While each of these studies considered socio-demographic variables, none explored the effects of different socioeconomic variables in combination.

We used cluster analysis to asses the effects of the combined socio-demographic variables that were identified in the review of literature on child obesity (measured as BMI), nutrition knowledge, and eating behavior among students in Grade 3 (ages 8–10 years) in China.

## Methods

### Study design

The data used in this analysis were collected at the baseline for the evaluation of an innovative educational program designed to improve nutritional well-being of elementary school children. The program’s development involved technical support from the China Center for Health Education (Beijing), education and public health officials at the provincial level, and school administrators, teachers and parents at the local level. This was a multiyear project. Prior to the school health education program’s implementation all parents received information about the program and signed informed consent documents approving their children’s participation. The city Education Department’s health education professionals collected the data from students in their classrooms before and after the health education program implementation.

Multistage stratified cluster sampling was used for sample selection. First, two economically different provinces were chosen: one economically developed (Shandong) and one economically disadvantaged (Qinghai). Shandong is an east-coast province situated about halfway between Beijing and Shanghai. Shandong is one of the most populous provinces in China, with 95.8 million people, and is the third wealthiest province in China [20]. Qinghai province is in western China next to neighboring Tibet. Qinghai’s population of 5.6 million includes a number of ethnic minority groups and many ethnic autonomous areas. The economy is among the poorest in China [21].

In each province, cities fall into one of three classes, based on population size. To increase diversity of the sample, schools were chosen from cities of different classes. All students in Grade 3 (ages 8–10 years) in each school were included in the school health education program and evaluation. Each chosen school met three criteria: the school had never conducted a health education project or diet/nutrition-related project before; the total number of students enrolled in the school was over 1000; and leaders of the school and local health bureau approved the project and were willing to cooperate with the research team. The overall project included a baseline survey, an educational intervention, and a post-intervention survey. Only data from the baseline survey were used in this analysis.

### Measurements

The questionnaire that was completed by the students was developed by Chinese epidemiologists and educators, written in simplified Chinese and contained four parts.

#### Demographics

Home province, gender, age, parents’ educational level, main person cooking at home, whether living with parents, and frequency of living with grandparents.

#### Anthropometric information

Height and weight measures recorded by the school doctor were used to calculate Body Mass Index (BMI = weight/height*height). BMI results were according to the age-specific standards established by the China Ministry of Education [22]. A separate question asked students about their self-image: whether they felt they were obese, overweight, normal weight or underweight.

#### Nutrition knowledge questions

Questions assessed student’s knowledge about most important staples, fruits, vegetables, refined and unrefined grains, and daily water consumption. Correct answers were scored 1, to provide a score between 0 and 7, with 7 being highest number of correct answers.

#### Eating behaviors questions

Questions assessed behaviors including frequency of eating breakfast, choice of beverages, staple food for lunch and dinner, vegetable/meat proportions for lunch and dinner, frequency of eating vegetables, fruits and fried foods, and consumption of milk and water. Answers that met pre-established criteria (for example, ate breakfast “every day”) were scored 1, to provide a score from 0 to 9, with a 9 being the highest number of eating behaviors meeting the criteria for health.

Since nutrition knowledge and eating behavior were assessed with criterion reference tests, this precluded reliability estimates [23, 24].

Students in China are accustomed to providing family demographic information, on surveys, tests and exams beginning as early as age 6. As with any survey there is a likelihood of inaccurate information, but in this environment the likelihood is small. The demographic questions included an “I don’t know” option to allow students to indicate not knowing or to opt out of answering.

### Statistical analysis

Cluster analysis was chosen because the socio-demographic variables examined here are known to have high association with each other. We believed other methods, such as regression models, that focus on identification of independent effects and do not take into account the actual associations between the variables would provide misleading results. To analyze highly intercorrelated variables we preferred to examine their combined rather than their singular effects. Cluster analysis examines the joint effects of the variables. The members of each cluster are similar to each other in their characteristics and different from those in the other clusters. This method is especially applicable to socio-demographic variables for findings underlying patterns of commonality that may not be readily apparent from basic descriptive statistics or variable-centered analyses like correlation and regression.

Cluster analyses were performed with the Statistical Package for the Social Sciences (SPSS version 22.0) two-step cluster procedure using categorical variables. For binary categories (e.g., yes-no), categorical clustering maximizes the difference in the binary distributions of the groups. For multinomial categories, categorical clustering differentiates on the basis of response percentage within the response options. SPSS cluster procedure default options of Log-likelihood as the measure for cluster distance for pre-clustering, and the Bayesian Information Criterion (BIC) was the clustering criterion for the clustering step. SPSS automatic cluster determination was used to produce the initial cluster solution. Then alternative cluster solutions around this initial solution were tested for potentially better fits using three criteria: (a) silhouette, which measures how tightly clusters group, with higher scores indicating a better fit; (b) sums of squares within group (SSE), which measures cohesions within clusters, with lower scores indicating a better fit; and (c) sums of squares between group (SSB), which measures separation, with higher scores indicating a better fit. Missing data was handled with list wise deletion.

The cluster analysis was done with socio-demographic variables identified in the literature review [10–19]: gender, father’s education, mother’s education, whether student lived with parents, frequency of student living with grandparents, main person cooking at home, and self-image. Descriptive statistics highlighted the characteristics of the clusters.

Subsequent Analysis of Variance (ANOVA) was used to compare the differences among clusters in BMI, nutritional knowledge and eating behaviors. The student knowledge scores and behavior scores, which are numeric counts of how many criteria a student met, were treated as continuous ratio-level numbers for the ANOVA.

## Results

### Cluster profile

Of the 3922 completed surveys 396 (10%) were excluded from the analysis due to missing values. The SPSS two-step automatic cluster determination identified a four-cluster solution. We then tested alternative two-, three-, and five-cluster solutions. The four-cluster solution had the best silhouette goodness of fit consistent with the automatic cluster determination fit (4-cluster = .180; 5-cluster = .162; 3-cluster = .158; 2-cluster = .168). The four cluster solution had the second best SSE (4-cluster = .146; 5-cluster = .141; 3-cluster = .158; 2-cluster = .165) and second best SSB (4-cluster = .037; 5-cluster = .041; 3-cluster = .025; 2-cluster = .017) goodness of fit indicators. Although the five-cluster solution had the best SSE and SSB indicators, it had the second lowest silhouette value and was considerably lower than the four-cluster solution. Overall the four-cluster solution had the best aggregate goodness of fit, so it was chosen as the cluster solution.

Values for the clustering variables for the four clusters are shown in Table 1. Four characteristics (frequency of living with grandparents, main person doing the cooking at home, mother’s education, and father’s education) were identified as the most important variables for distinguishing the four clusters. The other four characteristics (province, gender, whether living with parents, and body self-image) did not distinguish the clusters. The results showed that the students who lived with grandparents all had their grandparents as the main cook at home. So in our description of clusters, living with grandparents and who cooked at home were combined under the heading of family type.

**Table 1:** Frequency distribution of socio-demographic characteristics among clusters (*N* = 3526)

Socio-demographic characteristic	*Cluster 1* (two-gen/ low-ed)^a^		*Cluster 2* *(two-gen/ high-ed)* ^*b*^		*Cluster 3* (two-gen/ med-ed)^*c*^		*Cluster 4* (three-gen/ high-ed)^*d*^	
	*n* = 858		*n* = 1072		*n* = 972		*n* = 624	
	*n*	*%*	*n*	*%*	*n*	*%*	*n*	*%*
Province								
Shangdong	491	57.2	722	67.4	675	69.4	384	61.5
Qinghai	367	42.8	350	32.6	297	30.6	240	38.5
Gender								
Male	438	51.0	564	52.6	514	52.9	363	58.2
Female	420	49.0	508	47.4	458	47.1	261	41.8
Education level (father)^e^								
College or higher	124	14.5	806	75.2	286	29.4	362	58.0
Senior middle	250	29.1	207	19.3	537	55.2	192	30.8
Junior middle	484	56.4	59	5.5	149	15.3	70	11.2
Education level (mother)^e^								
College or higher	11	1.3	1072	100.0	0	0	376	60.3
Senior middle	4	0.5	0	0	972	100.0	181	29.0
Junior middle	843	98.3	0	0	0	0	67	10.7
Living with parents								
Yes	723	84.3	965	90.0	873	89.8	417	66.8
No	135	15.7	107	10.0	99	10.2	207	33.2
Living with grandparents^e^								
Yes	93	10.8	0	0.0	21	2.2	494	79.2
Occasionally	420	49.0	673	62.8	603	62.0	93	14.9
No	345	40.2	399	37.2	348	35.8	37	5.9
Who mainly cooks^e^								
Mother	667	77.7	800	74.6	730	75.1	121	19.4
Father	107	12.5	217	20.2	171	17.6	35	5.6
Grandmother	59	6.9	8	0.7	47	4.8	407	65.2
Grandfather	8	0.9	14	1.3	11	1.1	35	5.6
Nannie	8	0.9	23	2.1	12	1.2	12	1.9
Others	9	1.0	10	0.9	1	0.1	14	2.2
Self-reported body weight								
Very thin	66	7.7	61	5.7	71	7.3	34	5.4
A little thin	221	25.8	281	26.2	249	25.6	134	21.5
Normal	399	46.5	539	50.3	477	21.5	338	54.2
A little fat	151	17.6	172	16.0	162	16.7	106	17.0
Very fat	21	2.4	19	1.8	13	1.3	12	1.9

^a^(two-gen/low-ed) —two-generation family, low-education parents (9 years or less of school); ^b^(two-gen/high-ed)—two-generation family, high-education parents (college or higher); ^c^ (two-gen/med-ed).—two-generation family, medium-education parents (10–12 years of school); ^d^(three-gen/high-ed) —three-generation family, high-education parents (college or higher); ^e^Distribution between four clusters is statistically significant

Cluster 1 was characterized by two-generation families and parents with low education (two-gen/low-ed). This cluster included 858 students (24%). Parents’ education was mainly junior middle school or lower (9 years of education or less: mothers 98.3%, father’s 56.4%). Mother was the main person cooking at home (77.7%). The student lived with the grandparents only occasionally (49.0%).

Cluster 2 was characterized by two-generation families and parents with high education (two-gen/high-ed). This cluster included 1072 students (30%). Parents education was college or higher (mother’s 100.0%, father’s 75.2%). Mother was the main person cooking at home (74.6%). The student lived with the grandparents only occasionally (62.8%).

Cluster 3 was characterized by two-generation families and parents with medium education (two-gen/med-ed).This cluster included 972 students (28%). Parents’ education was senior middle school or equal (10–12 years of education: mother’s 100.0%, father’s 55.2%). Mother was the main person cooking at home (75.1%). The student lived with the grandparents only occasionally (62.0%).

Cluster 4 contained three-generation families with higher educated parents (three-gen/high-ed). This cluster included 624 students (18%). Parents’ education was college or higher (mother’s 60.3%, father’s 58.0%). Grandmother was the main person cooking at home (65.2%). The students frequently lived with the grandparents (79.2%).

### BMI differences

There was no significant difference in BMI scores between the students in the four clusters as determined by one-way ANOVA (*F*(3,3425) = 0.728, *p* = 0.535) (Table 2).

**Table 2:** One-way ANOVA on BMI, nutrition knowledge, and eating behaviors (Mean ± *SD* or *n*(%))

	Cluster 1 (two-gen/low-ed)		Cluster 2 (two-gen/high-ed)		Cluster 3 (two-gen/med-ed)		Cluster 4 (three-gen/high-ed)		*F*	*p*
	Mean	*SD*	Mean	*SD*	Mean	*SD*	Mean	*SD*		
BMI	17.20	4.40	17.50	4.97	17.27	4.53	17.38	4.72	0.73	0.535
Knowledge^a^	3.09	1.46	3.46	1.48	3.34	1.51	3.46	1.49	12.05	0.000**
Behavior^b^	3.69	1.53	4.17	1.76	3.93	1.66	4.12	1.69	14.82	0.000**

*SD* Standard Deviation

*p < 0.05

**p < 0.001

^a^Total score = 7

^b^Total score = 9

### Nutrition knowledge

Table 2 shows a significant difference in nutrition knowledge between the four clusters (*F*(3,3522) = 12.052, *p* = 0.000). A Tukey post-hoc test indicated the nutrition knowledge was higher in Cluster 2 (two-gen/high-ed) (−0.372, *p* = 0.000), Cluster 3 (two-gen/med-ed) (MD = −0.249, *p* = 0.002) and Cluster 4 (three-gen/high-ed) (MD = −0.377, *p* = 0.000) compared to Cluster 1 (two-gen/low-ed) There were no differences in children’s nutrition knowledge scores between Cluster 2 and Cluster 3, between Cluster 2 and Cluster 4, and no difference between the Cluster 3 and 4.

### Eating behavior

Table 2 shows a significant difference in children’s eating behaviors scores between the four clusters (*F*(3,3522) = 14.818, *p* = 0.000). A Tukey post-hoc test showed that eating behavior scores were significantly higher in Cluster 2 (two-gen/high-ed) (MD = −0.476, *p* = 0.000), Cluster 3 (two-gen/med-ed) (MD = −0.236, *p* = 0.013) and Cluster 4 (three-gen/high-ed) (MD = −0.422, *p* = 0.000) compared to Cluster 1 (two-gen/low-ed). The eating behaviors scores were significantly higher in Cluster 2 (two-gen/high-ed) (MD = −0.240, *p* = 0.006) compared to Cluster 3 (two-gen/med-ed). There were no differences between Cluster 2 and Cluster 4, and between Cluster 3 and Cluster 4.

## Discussion

This study examined a number of socio-demographic characteristics related to obesity in combination, something not previously done in nutrition studies of this type in China. Analysis based on these characteristics identified four clusters.

Clusters differed most strongly based on family structure, with three of the four clusters having similar two-generation families with the mother doing most cooking. The fourth cluster (three-gen/high-ed) was characterized by a three-generation family structure with grandparents living in the home or the student living solely with the grandparents, and grandmother acting as the main cook. Despite the clear difference in family structure between the three-generation family and the other three clusters of two-generation family, the family structure differences did not lead to differences in nutrition knowledge, eating behaviors, or BMI.

Instead, primary school children’s nutrition knowledge and eating behavior most differed by levels of parental education, with the students of parents with the lowest education level (Cluster 1) scoring the lowest in nutrition knowledge and eating behaviors. Students of the two clusters with high parent education level (Cluster 2 two-generation household and Cluster 4 three-generation household) had higher nutrition knowledge scores and higher eating behavior scores than students of parents with medium education level (Cluster 3), but the difference was not significant. These findings suggest a strong effect of parental education on their children’s nutrition knowledge and eating behavior, with a clear advantage for children whose parents attain at least a high school education (10–12 years of education).

The selection of one economically developed province and one economically undeveloped province for this project reflected an assumption that economic development would be an important factor in childhood obesity; but home province did not contribute strongly to creating the clusters described here relative to the other characteristics examined. However the distribution of students from the two provinces within clusters was significantly different. Our data showed that Cluster 1 (two-gen/low-ed) had the highest proportion of students from Qinghai, the least economically developed province.

The absence of significant differences in BMI for children in Cluster 1 was unexpected. However, BMI scores among children this young are known to sometimes be unreliable [25]. If the children in the clusters were equally physically active, physical activity may override the effects of knowledge, eating, or parental education on BMI.

These results need to be seen in the context of China’s economic development. In the future, there will likely be fewer three-generation families as employment opportunities lure parents away from their hometowns. More young children will spend time in daycare arrangements or live with grandparents, decreasing the beneficial effect that higher parental education has on child eating behaviors and nutrition knowledge. For the children of parents with less education, there will be no change. However, the improving economic conditions in China are leading to an increase in the number of years that people attend school; nevertheless, any benefit to children’s nutrition, based on parent education, is a generation away. Economic improvements and increasing employment opportunities may decrease the time available to cook in the home and increase likelihood for families and children to eat out at restaurants. Increasingly, prepackaged and less healthy foods are available [5, 6].

There will be those who advocate for school-based nutrition education even in the primary school level. But the potential for traditional classroom-based programs to affect younger children’s body weight is limited, as all the decisions related to eating are managed by the parents or someone else in the home. Viewed in a holistic way, however, school-based nutrition education that includes multiple education inputs and environmental changes has a real potential to be an effective part of a national policy to reduce obesity. For example, Qian and colleagues [26], in a two-year elementary school-based obesity-reduction intervention, increased students knowledge, improved attitude, and improved nutrition behavior, and reduced BMI’s, mostly among boys. Their project was based on WHO’s Health Promoting School Model [27], which included classes for parents on the importance of healthy food selection and physical activity, in addition to school health promotion activities for teachers, administrators, building maintenance employees, and the installation of playground equipment. Qian et al.’s findings [26], along with the results of this analysis, suggest that the effectiveness of school-based nutrition- and obesity-related programs for children could be increased by adding a parent/family nutrition education component, especially for schools in less economically developed areas where there is a larger proportion of parents having less than a high school education.

### Limitations

This study was the first to use a cluster analysis to look for socio-demographic characteristics that relate to the nutrition behavior of Chinese urban primary students. We used the results from the few available studies in the literature and the descriptive data collected as part of this study to derive our indicators, but the scarcity of previous studies is a limitation. The socio-demographic characteristics chosen reflected the best available thinking at the time, but clearly there are other variables that might be explored. Collecting reliable information from young children is difficult but considerable care and training preceded data collection. Chinese students, even at a young age are used to taking tests, examinations and surveys and providing demographic information about themselves. There was little reason to believe students were not responding to questions in the survey accurately. The number of schools was small, but schools were selected to be representative of the two provinces. Despite procedures to help insure objectivity, the presence of the expert team from Beijing and their direct involvement in many phases of this project may have introduced biases that could affect the external validity of the results. The ANOVA analysis used a numeric count of how many criteria a student met; therefore, the counting universe for these measures was censored, as there wasn’t an unlimited number of possible criteria. Table 2, shows the distributions are quasi-normal with no particular skewness. As ANOVA is highly robust to even large violations of normality, the obtained scores we feel were suitable for this analysis. As a field-based project, the results are valuable in planning similar future projects and in providing actual data with which to inform policy discussions.

## Conclusions

Parental educational level is important for elementary-school-aged children’s nutritional knowledge. But, importantly, student nutrition knowledge seemed to benefit about equally as long as the educational level of their parents is high school or higher (10–12 years of school or more). There doesn’t appear to be any additional effect from parents having a college education. For students’ correct eating behaviors, however, there was more evidence that the parents’ educational level had a dosage effect, with student’s eating behavior scores increasing with each higher level of parental education. Parents’ educational level should be considered when designing interventions to improve the nutritional knowledge and eating behavior of primary school students.
